# Improving Trainee Engagement in Trainee Council Meetings at Birmingham and Solihull Mental Health Foundation Trust

**DOI:** 10.1192/bjo.2025.10342

**Published:** 2025-06-20

**Authors:** Sarah Brennan, Vicki Ibbett, Ruth Scally

**Affiliations:** Birmingham and Solihull Mental Health Foundation Trust, Birmingham, United Kingdom

## Abstract

**Aims:** Trainee Council Meetings (TCMs) offer a dedicated time and place for trainees working in the Trust to highlight issues and raise concerns relating to their rotations.

Trainee engagement in these meetings has historically been variable. This project was developed with the aim of improving trainee engagement with TCMs. It is part of a larger ‘Raising Concerns’ Quality Improvement Project within the Trust.

Increase attendance at Trainee Council Meetings (TCMs) by Foundation, GP and Core Psychiatry Trainees.

Improve structure and organisation of Trainee Council Meetings.

Improve trainee access to records of meeting minutes.

**Methods:** Retrospective TCM attendance data was collected in Summer 2023. The only data available were numbers of attendees, not trainee grade. The following issues were identified:

TCMs sometimes took place in person and sometimes took place online (via Microsoft Teams). Attendance tended to be poorer for in-person meetings than those online.

There was no clear leadership structure within the Trainee Council.

There was lack of clarity over which representatives were responsible for planning and facilitating TCMs. This led to an unfair and unequal distribution of TCM workload.

The following change ideas were implemented from the respective dates:

July 2023 – It was agreed that TCMs would always take place online.

August 2023 – Development of two leadership roles within the Trainee Council: ‘Trainee Representative and Induction Co-ordinators’ and ‘SHO Inclusion Co-ordinators’. Planning and facilitation of TCMs was agreed as a responsibility to be shared amongst these representatives.

Development of a Meeting Proforma (see Appendix), clarifying actions to be taken by council leads before, during and after TCMs.

Attendance data were collected prospectively between September 2023 and March 2024. Data included numbers of trainees attending and trainee grade.

**Results:** Trainee attendances before change ideas:

December 2022 – 20

March 2023 – 9

June 2023 – 22

Trainee attendances after change ideas:

September 2023 – 38

November 2024 – 34

March 2024 – 36

**Conclusion:** Attendance data show that there has been an improvement in numbers of trainees attending Trainee Council Meetings following implementation of change ideas.

Attendance of trainees by grade was unknown prior to June 2023. The majority of attendees between September 2023 and March 2024 were Core Psychiatry trainees. Attendance by Foundation and GP trainees is low.

